# Association Between *VKORC1* Gene Polymorphisms and Osteopenia and Osteoporosis: A Systematic Review and Meta-Analysis

**DOI:** 10.3390/medicina62010180

**Published:** 2026-01-15

**Authors:** Ştefan Cristian Vesa, Vlad-Mihai Ichim, Silvina Iluț, Stefano Miglietta, Mihai Lupu, Camelia Alexandra Coada, Antonia Eugenia Macarie, Ovidiu Chiroban, Anca Dana Buzoianu, Octavia Sabin

**Affiliations:** 1 Division of Pharmacology, Toxicology and Clinical Pharmacology, Department of Morpho-Functional Sciences, University of Medicine and Pharmacy “Iuliu Hațieganu”, 8 Victor Babeş Street, 400347 Cluj-Napoca, Romaniaabuzoianu@umfcluj.ro (A.D.B.); octavia.sabin@umfcluj.ro (O.S.); 2 Faculty of Medicine, University of Medicine and Pharmacy “Iuliu Hațieganu”, 8 Victor Babeş Street, 400347 Cluj-Napoca, Romania; ichim.vlad.mihai@elearn.umfcluj.ro; 3 Department of Medical Disciplines, Faculty of Nursing and Health Sciences, University of Medicine and Pharmacy “Iuliu Hațieganu”, 8 Victor Babeş Street, 400347 Cluj-Napoca, Romania; 4 Department of Biosciences, Biotechnologies and Environment, University of Bari, 70125 Bari, Italy; 5 Division of Physiology, Department of Morpho-Functional Sciences, University of Medicine and Pharmacy “Iuliu Hațieganu”, 8 Victor Babeş Street, 400347 Cluj-Napoca, Romania; 6 Division of Geriatrics and Gerontology, Department of Medical Specialties, University of Medicine and Pharmacy “Iuliu Hațieganu”, 8 Victor Babeş Street, 400347 Cluj-Napoca, Romania; 7 Division of Forensic Medicine, Department of Community Medicine, University of Medicine and Pharmacy “Iuliu Hațieganu”, 8 Victor Babeş Street, 400347 Cluj-Napoca, Romania

**Keywords:** osteoporosis, bone mineral density, vitamin K epoxide reductases, VKORC1 protein, genetic association studies, postmenopause/metabolism

## Abstract

*Background and Objectives:* The vitamin K epoxide reductase complex subunit 1 (VKORC1) plays a central role in the vitamin K cycle, which is essential for γ-carboxylation of multiple bone-related proteins. Genetic variants in *VKORC1* may influence bone mineral density (BMD) and osteoporosis risk. *Materials and Methods:* A systematic review and meta-analysis were conducted to evaluate the association between *VKORC1* polymorphisms and osteopenia and osteoporosis. Relevant studies were identified through PubMed, Scopus, and Web of Science databases. Data on study characteristics, genotypes, BMD measurement, ethnicity, sex, and menopausal status were extracted. *Results*: Six studies comprising 7335 participants were included. All studies assessed BMD using dual-energy X-ray absorptiometry (DXA). The mean participant age ranged from 41.9 to 63.7 years. The *VKORC1* variants most frequently studied, which were included in the meta-analysis, were rs9923231 and rs9934438. The overall effect of *VKORC1* risk alleles on osteopenia/osteoporosis was significant with a *p* = 0.041 (fixed effects OR = 1.16, 95% CI = 1.01–1.35). Heterogeneity among studies was insignificant *(I*^2^ = 0%, *p* = 0.893). *Conclusions:* A modest association was observed for the *VKORC1* variants. The current body of evidence requires further studies to elucidate whether *VKORC1* polymorphisms have a clinically meaningful role in bone health.

## 1. Introduction

Osteoporosis is a highly prevalent disease representing a major global health problem [1,2]. It is characterized by low bone mass and microarchitectural deterioration of bone matrix, resulting in increased bone fragility and fracture risk [1,2], impacting more frequently postmenopausal women and the elderly population. With the aging global demographic, the burden of osteoporosis-related morbidity, mortality, and healthcare costs continues to rise [2]. Understanding the molecular and genetic determinants of bone metabolism and implementing efficient prevention strategies has therefore become a central goal in bone research [1,2,3].

Bone mineral density (BMD), the most widely used quantitative measure for diagnosing osteoporosis, is influenced by a complex interplay of genetic, hormonal, nutritional, and environmental factors [4]. Multiple genetic factors have been identified as contributing to bone health and osteoporosis susceptibility, including multiple candidate genes involved in calcium metabolism, collagen formation, estrogen signaling, and vitamin D pathways [4,5]. Vitamin K plays a fundamental role in bone physiology by participating in the γ-carboxylation of specific glutamate residues in bone matrix proteins such as osteocalcin (OC) and matrix Gla protein (MGP) [6]. Carboxylated OC binds hydroxyapatite crystals and contributes to bone strength and mineralization, while undercarboxylated OC, associated with vitamin K deficiency or impaired recycling, is linked to reduced BMD and increased fracture risk [6,7]. The VKORC1 enzyme catalyzes the reduction of vitamin K epoxide back to its active hydroquinone form, thus maintaining the vitamin K cycle. Genetic variants that alter *VKORC1* expression or enzyme efficiency could potentially influence the carboxylation status of osteocalcin and other vitamin K-dependent proteins, providing a plausible biological mechanism linking *VKORC1* polymorphisms to bone health. This line of thought stems from the well-known impact of *VKORC1* gene variants on the response to anticoagulant therapy with vitamin K antagonists such as warfarin. In fact, this effect is clinically relevant enough to have been included in guidelines such as the Clinical Pharmacogenetics Implementation Consortium (CPIC) Guideline for Pharmacogenetics-Guided Warfarin Dosing [8] and the Dutch Pharmacogenetics Working Group (DPWG) [9]. On this note, the most relevant single-nucleotide polymorphisms (SNPs) in *VKORC1* are rs9923231 (−1639 G>A), rs9934438, and rs8050894, which have been shown to affect *VKORC1* transcription and thus warfarin sensitivity [10]. These same variants may also modulate bone turnover through altered vitamin K metabolism. However, results from individual association studies on *VKORC1* SNPs and BMD have been inconsistent. Some have demonstrated a significant correlation between VKORC1 polymorphisms and lower BMD or increased osteoporosis risk, while others have failed to detect such associations, potentially due to small sample sizes, population heterogeneity, or differences in menopausal status and ethnic background.

Given these conflicting findings, and that osteopenia/osteoporosis is a polygenic disease in which individual genetic variants are expected to have small effects, a comprehensive evaluation of underpowered studies is needed to determine if VKORC1 gene polymorphisms influence bone health, and to which extent. Therefore, the present systematic review and meta-analysis aimed to summarize and assess the available evidence linking *VKORC1* gene polymorphisms (rs9934438 and rs9923231) with bone mineral density, and osteopenia and osteoporosis risk. By integrating data from multiple studies, we sought to clarify whether *VKORC1* polymorphisms contribute to osteopenia and osteoporosis susceptibility and to explore the potential biological and clinical implications of these findings.

## 2. Materials and Methods

### 2.1. Systematic Article Search Strategy

Databases searched included PubMed, Scopus, and Web of Science (last updated 1 September 2025). Search terms included “VKORC1,” “vitamin K epoxide reductase”, “polymorphism”, “bone mineral density”, “osteopenia”, “osteoporosis”. References of the articles reviewed in full text were also examined for backward citation searching, and additional searches were performed manually in gray literature sources such as Google Scholar. This systematic review follows the PRISMA guidelines [11] and was registered in PROSPERO with the code CRD420251178664.

### 2.2. Inclusion Criteria and Study Selection

This work included human studies that investigated the association between *VKORC1* SNPs and BMD or osteopenia or osteoporosis. Eligible studies were required to: (i) provide data on genotype distributions across control and study groups; (ii) provide odds ratios associated with *VKORC1* allele burden; (iii) assess BMD using dual-energy X-ray absorptiometry (DXA). No restrictions for sex, ethnicity or article language were imposed. Studies were excluded if: (i) insufficient data was provided for data extrapolation and pooled analysis; (ii) measured BMD using other methods than DXA or did not specify the method; (iii) studies on animal models or in vitro models; (iv) conference abstracts, editorials, review articles.

### 2.3. Data Extraction

From each study, we extracted data comprising author, publication year, sample size, demographic characteristics, menopausal status, *VKORC1* variant(s) investigated, and key results (Table 1).

### 2.4. Quality Assessment

Each study was assessed using the Newcastle–Ottawa Scale [12] for genetic association studies. Results were reported for each question and chapter in the Supplementary Materials.

### 2.5. Statistical Analysis

All statistical analyses were performed using the R statistical software [13] (version 4.4.3 2025-02-28 ucrt, “Trophy Case”) with the *meta* package [14]. Osteopenia- and osteoporosis-affected patients were both considered for the study group, while healthy individuals were considered for the control group. Effect sizes were expressed as odds ratios (ORs) with corresponding 95% confidence intervals (CIs) to estimate the association between each *VKORC1* polymorphism and the risk of osteopenia or osteoporosis. For each included study, the logarithm of the odds ratio (logOR) and its standard error were used as input parameters. Pooled estimates were calculated using both fixed-effect and random-effects models. Given the expected methodological and population variability across studies, the random-effects model was considered the primary analysis.

Statistical heterogeneity was assessed using the Cochran’s Q test and I^2^ statistic, with *p* < 0.10 for the Q test or I^2^ > 50% indicating substantial heterogeneity. When I^2^ was 0%, a fixed-effect and random-effects model yielded identical results, confirming the robustness of the findings. Forest plots were generated to visualize the individual and pooled ORs for each polymorphism. Potential publication bias was assessed visually through funnel plots and using Egger’s regression test. All statistical tests were two-sided, and *p* < 0.05 was considered statistically significant.

## 3. Results

### 3.1. Article Screening and Selection

**Figure 1:**
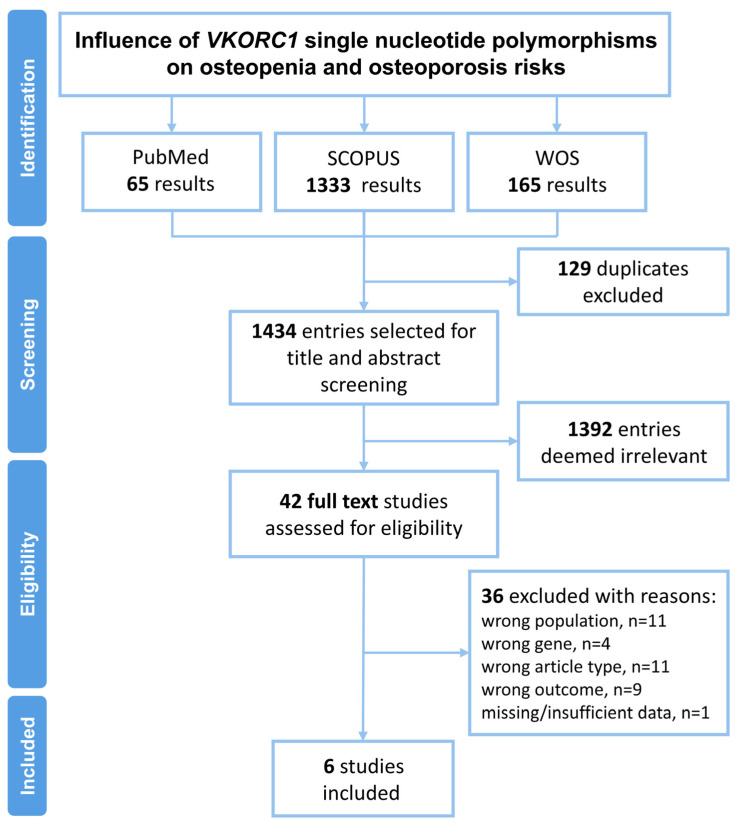
PRISMA workflow for the identification and selection of the articles.

A total of 1563 records were identified through database searching, including 65 from PubMed, 1333 from Scopus, and 165 from Web of Science (WOS). After removing duplicates, 1434 unique entries were retained for title and abstract screening. Following this step, 42 studies were retrieved and assessed for full-text eligibility. After applying the inclusion and exclusion criteria, a total of 6 studies were deemed eligible and were included in the meta-analysis evaluating the association between *VKORC1* SNPs and the risk of osteopenia and osteoporosis (Figure 1).

### 3.2. Study Characteristics

A total of five studies (2010–2021) with 7335 participants were included, comprising 1251 healthy volunteers with normal BMD, 252 patients with osteopenia and 772 patients with osteoporosis (Table 1 and Table S1). All BMD measurements were performed using dual-energy X-ray absorptiometry (DXA). Sample sizes ranged from 216 to 5160. Most studies were conducted on women with the majority being postmenopausal women, except in the Crawford et al. and Fodor D et al. studies which included both sexes. Moreover, the Crawford et al. study conducted subgroup analyses considering both sexes and ethnic groups.

The SNPs investigated were rs9923231, rs9934438, rs9934488, rs2359612, rs8050894, rs2884737, and rs7294 (Table 1 and Table S1). Since not all studies examined all polymorphisms, and only rs9934438 and rs9923231 were analyzed in more than one study, the meta-analysis was conducted on these two SNPs. Of note, the subgroups analyzed in the study of Crawford et al. were kept separate, as reported in their manuscript. Analyses were also performed with a previous pooling of these subgroups, obtaining consistent results in significance and heterogeneity indices.

**Table 1:** Summary of the included studies.

Author, Year	*VKORC1* Gene Variant	N Total Patients: N Controls/Osteopenia/Osteoporosis	Ethnicity	Mean Age	Sex M/F # F at Menopause	Summary of Main Results
Ciubean et al., 2019 [15]	rs9934438	364: 136/0/228	Romanian	63.45 ± 8.16 65.5 ± 7.39	0/364 # 364	No significant association was found.
Crawford et al., 2010 [16]	rs9923231, rs9934438, rs2359612, rs8050894, rs2884737, rs7294	5668: 738/0/4930	Non-Hispanic white; Non-Hispanic black; Mexican-American	41.92	2298/2862 # 459	Significant association found in non-Hispanic black males.
Fodor et al., 2013 [17]	rs9934438	239: 66/105/68	Romanian	63.7 ± 9.09	35/204 # n/a	Significant differences obtained by comparing CC + CT and TT genotypes.
Jin He, 2021 [18]	rs9923231, rs9934488	606: 318/0/288	Han Chinese	61.07 ± 6.10	0/606 # 288	Statistically significant link between rs9923231 and osteoporosis.
Kutluturk et al., 2018 [19]	rs9923231	216: 140/0/176	Turkish	57.87 ± 6.54	0/216 # 216	No statistically significant results could be proven.
Taha et al., 2023 [20]	rs9934438 rs3736228 rs2297480 rs1234612	750: 591/147/12	Saudi Arabic	18–40 *	0/750 # 0	An increased OR for those with gene variants rs3736228 and rs2297480 for osteopenia.

* range. Abbreviations: VKORC1—vitamin K epoxide reductase; rs—Reference Single Nucleotide Polymorphism; N—number; M—male; F—female; OR—odds ratio; #—number of.

### 3.3. rs9923231 and rs9934438 Impact on the Risk of Osteopenia and Osteoporosis

**Figure 2:**
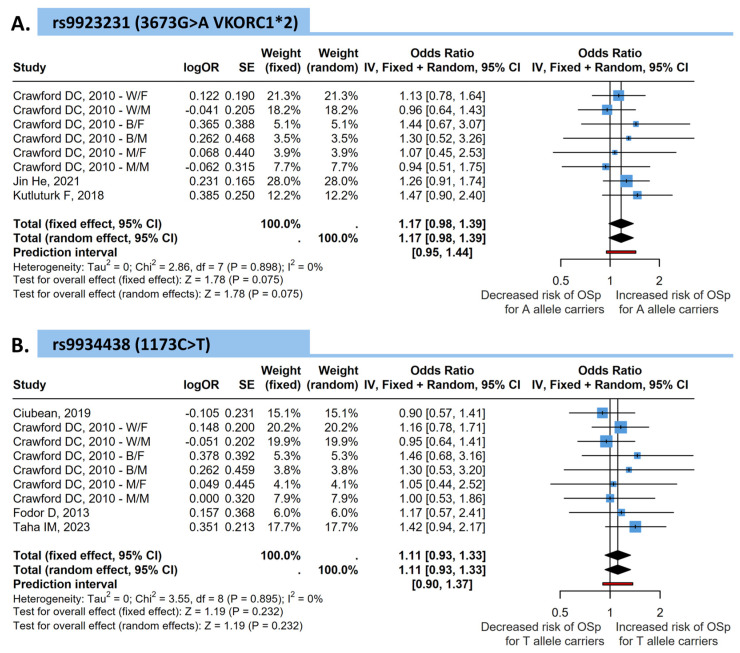
Forest plots showing the association between *VKORC1* polymorphisms and the risk of osteopenia or osteoporosis (OSp). (**A**) VKORC1 rs9934438 (1173C>T; C6484T) and (**B**) *VKORC1* rs9923231 (−1639G>A; 3673G>A; VKORC1*2). Pooled odds ratios (ORs) and 95% confidence intervals (CIs) were calculated using fixed- and random-effects models. Squares represent individual study effect sizes, and the diamond represents the pooled estimate [15,16,17,18,19,20].

The pooled analysis from eight comparisons demonstrated no significant association between the *VKORC1* rs9923231 polymorphism and osteopenia/osteoporosis risk. The fixed-effects pooled OR was 1.17 [95% CI: 0.98–1.39, *p* = 0.075], showing only borderline significance for A allele carriers to have an altered risk of bone loss compared to non-carriers. Heterogeneity among studies was minimal (*I*^2^ = 0%, *p* = 0.898) (Figure 2).

Pooled analysis of nine comparisons showed no significant association between the *VKORC1* rs9934438 polymorphism and the risk of osteopenia or osteoporosis. The overall pooled OR under a fixed-effects model was 1.11 [95% CI: 0.93–1.33, *p* = 0.232], indicating no difference in risk between T allele carriers and non-carriers. Heterogeneity across studies was low (*I*^2^ = 0%, *p* = 0.895), suggesting consistency among studies (Figure 2).

### 3.4. Subgroup Analysis on Female Participants

Given the well-established effects of estrogen and progesterone, and the absence of these hormones following menopause, a sub-analysis considering only female participants was conducted with the purpose to explore potential sex-specific effects.

**Figure 3:**
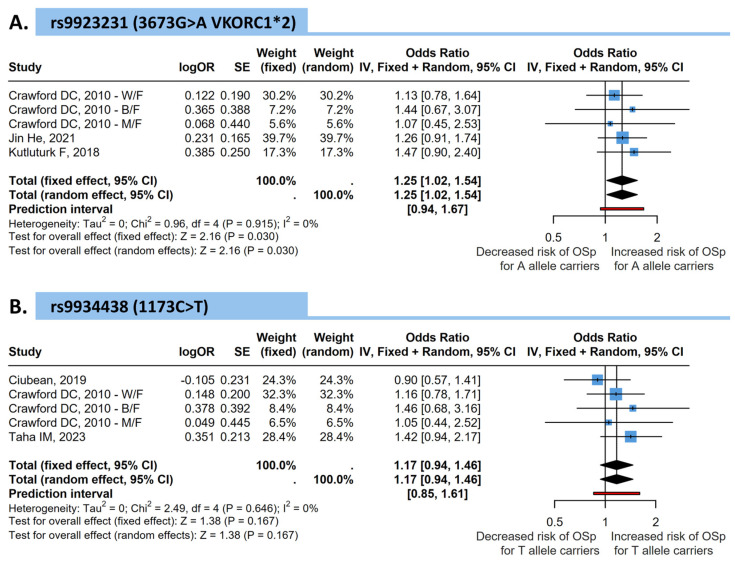
Forest plots showing the association between *VKORC1* polymorphisms and the risk of osteopenia or osteoporosis (OSp) considering only female participants. (**A**) *VKORC1* rs9934438 (1173C>T) and (**B**) *VKORC1* rs9923231 (3673G>A; VKORC1*2). Pooled odds ratios (ORs) and 95% confidence intervals (CIs) were calculated using fixed- and random-effects models. Squares represent individual study effect sizes, and the diamond represents the pooled estimate. W/F: Non-Hispanic whites/Females; B/F: Non-Hispanic blacks/Females; M/F: Mexican-Americans/Females [15,16,18,19,20].

For the *VKORC1* rs9923231 (3673G>A) polymorphism, the pooled analysis suggested a significant association, with carriers of the A allele showing an increased risk of osteopenia or osteoporosis (random-effects OR = 1.25, 95% CI = 1.02–1.54, *p* = 0.030). Heterogeneity was low (*I*^2^ = 0%, *p* = 0.915) (Figure 3).

In contrast, for the *VKORC1* rs9934438 (1173C>T) polymorphism, the pooled analysis of five comparisons showed no significant association with osteopenia or osteoporosis risk among women (random-effects OR = 1.17, 95% CI = 0.94–1.46, *p* = 0.167). Heterogeneity was minimal (*I*^2^ = 0%, *p* = 0.646), indicating consistency among studies (Figure 3).

### 3.5. Pooled Meta-Analysis of the VKORC1 Risk Alleles Impact on the Osteopenia and Osteoporosis

**Figure 4:**
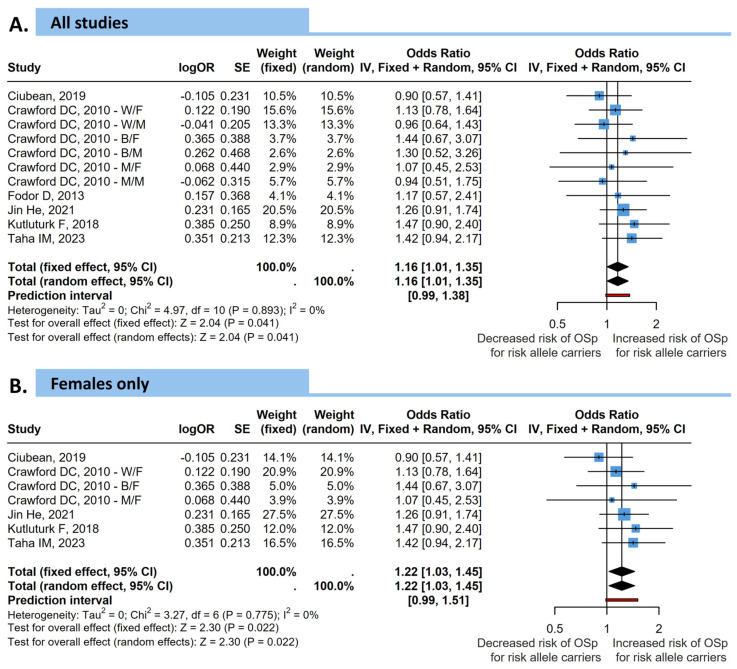
Forest plots showing the association between the burden of *VKORC1* risk allele (rs9923231 or rs9934438—a marker for rs9923231) and the risk of osteopenia or osteoporosis (OSp) on (**A**) all subjects and (**B**) considering only female participants. Pooled odds ratios (ORs) and 95% confidence intervals (CIs) were calculated using fixed- and random-effects models. Squares represent individual study effect sizes, and the diamond represents the pooled estimate. W/F: Non-Hispanic whites/Females; B/F: Non-Hispanic blacks/Females; M/F: Mexican-Americans/Females; W/M: Non-Hispanic whites/Males; B/M: Non-Hispanic blacks/Males; M/M: Mexican-Americans/Males [15,16,17,18,19,20].

Carriers of the rs9923231 A variant allele have consistently been reported to require a lower initial dose of warfarin than G allele carriers resulting in this promoter region variant being considered a causative SNP for low-dose warfarin phenotype [21]. *VKORC1* rs9934438 is in almost perfect linkage disequilibrium with rs9923231, as confirmed also by allele frequencies in multiple genetic ancestry groups [22]. Although functionally inert, rs9934438 is still used as a SNP marker for rs9923231 [21,23]. Given these factors, we considered it appropriate to conduct a pooled analysis of both SNPs in order to increase the power of the meta-analytical results (Figure 4).

When considering all studies conducted on both sexes, the overall effect of *VKORC1* risk alleles on osteopenia/osteoporosis resulted significant with a *p* = 0.041 (fixed effects OR = 1.16, 95% CI = 1.01–1.35). Heterogeneity among studies was insignificant (*I*^2^ = 0%, *p* = 0.893). Similarly, when the sub analysis was conducted using only female participants, the results remained significant with a *p*-value of 0.022 (fixed effects OR = 1.22, 95% CI = 1.03–1.45).

### 3.6. Publication Bias Analysis and Quality Assessment

Funnel plot and Egger’s test analysis showed no significant publication bias. Moreover, sunset enhanced funnel plot showed reduced power of the individual studies (less than 20%) (Supplementary Figure S1). Power analysis of the pooled results showed a cumulative power of this study of 100% to detect a significant effect should one be present.

The quality of the studies ranged from moderate to high, with NOS scores between 7 and 9 out of a maximum of 9 points. Most studies achieved high quality (≥8), while one was of moderate quality with a score of 7 (Supplementary Table S2).

Overall, studies demonstrated strong exposure analyses with validated PCR-based genotyping and clear case definitions through DXA-confirmed osteopenia/osteoporosis. However, there were consistent limitations in representativeness in terms of ethnicity, sexes, menopause status, and control of various confounder factors such as other parameters influencing bone health such as nutrition, vitamin D levels and supplement consumption. For example only Crawford et al. [16] used the NHANES III study making it nationally representative as well as adjusting for lifestyle (smoking, diet, hormone therapy, calcium, vitamin D, alcohol) and stratifying by sex and ethnicity. Few other studies accounted for dietary vitamin K intake, physical activity, or medication use (such as corticosteroids or warfarin) which are known to impact bone metabolism (Supplementary Table S2).

## 4. Discussion

To the best of our knowledge this is the first meta-analysis to examine the potential association between *VKORC1* SNPs and bone health outcomes, specifically osteopenia and osteoporosis. The results suggest a potential genetic link between *VKORC1* rs9923231 and rs9934438 variants and increased risk for osteopenia and osteoporosis.

The rs9923231 and rs8050894 polymorphisms, located in regulatory regions of the *VKORC1* gene, are associated with altered enzyme activity, with a well recognized impact on anticoagulant dosing [9,21]. Given the well-known role of vitamin K recycling and subsequent γ-carboxylation of bone matrix proteins, one might think that these SNPs could impair calcium binding and bone mineralization as well.

### 4.1. Summary of the Existing Literature

Although VKORC1 plays a central role in the vitamin K pathway and bone metabolism, the available evidence remains rather inconsistent and quite of low quality. First, it is worth noting that only few studies were identified. Most of them were limited by small sample sizes and heterogeneous populations. In fact, our power analysis of the individual studies confirmed that all had low statistical power (less than 20%), which substantially limits their ability to detect true associations, should one be present. This underpowered nature likely contributed to the lack of significant results in many of the included studies. This is a frequently seen problem in research and highlights the importance and need of good quality meta-analyses, which ensure a sufficient statistical power by pooling data from multiple studies and analyzing all patients together. In the present meta-analysis, despite including a small number of only six studies, the statistical power reached 100%, allowing for the detection of a significant association between *VKORC1* variants and the risk of osteopenia and osteoporosis. However, the magnitude of this effect was small (pooled OR = 1.16). This modest effect-size raises important questions about the clinical relevance and utility of *VKORC1* genotyping albeit statistically significant. Based on the current body of evidence, genetic testing for VKORC1 variants cannot yet be recommended as a reliable biomarker for osteoporosis risk prediction. Nevertheless, multigene panels which rely on analysis of various genes contributing to a phenotype might consider *VKORC1* in their risk scores.

In contrast, the clinical utility of *VKORC1* SNPs in anticoagulant therapy is well established. Variants in *VKORC1* significantly influence warfarin dose requirements, and genotype-guided dosing has been integrated into international pharmacogenetic guidelines [8,9,10]. However, the current meta-analysis suggests that this strong evidence base for anticoagulation cannot be extrapolated to bone metabolism, the impact of these SNPs remaining rather modest in nature. 

### 4.2. Other Roles of VKORC1 Polymorphisms on Bone Health

Interestingly, not all studies investigated the association between *VKORC1* polymorphisms and osteoporosis risk. Some other topics were tackled such as treatment response or association of the SNPs with the occurrence of fractures. For example, Ozgen et al. [24] evaluated the relationship between osteoporotic vertebral fracture and *VKORC1* SNPs (9041 G>A and 3673 G>A) in postmenopausal Turkish women. No significant results were found but again, this study was also conducted on a small sample size of only 150 women out of which 98 had at least one fracture. 

Next, Ciubean et al. [25] investigated the influence of *VKORC1* rs9934438 on alendronate response after 1 year and found no significant associations. Their sample size was severely small with only 25 postmenopausal women thus no significant conclusions can be drawn due to the limited power of the study.

Another study worth mentioning is that of Holzer et al. [26] who included males and females (22 subjects with normal BMD, 44 with osteopenia and 118 with osteoporosis) to investigate the impact of 3673G>A and the 9041G>A SNPs on BMD. Despite it being an initial candidate study for this meta-analysis, unfortunately it had to be excluded from the pooled analysis because of incomplete reporting which prevented the calculation of odds ratios, essential for a valid quantitative meta-analysis. They also could not provide any definitive results of their analyses, although they reported a trend towards the correlation of the 3673 variants with the circulating concentration of carboxylated Gla-OC (*p* = 0.07).

### 4.3. Limitations and Future Directions

While our findings are consistent with the established biological role of vitamin K in bone metabolism, several limitations should be acknowledged. The included studies often had relatively small sample sizes, heterogeneity in cohorts in terms of sexes, and variability among the included populations which limited subgroup analyses and resulted in insufficient statistical power. Only one study conducted subgroup analyses by ethnicity. Given that the allele frequencies of *VKORC1* polymorphisms differ across populations [22], their potential impact on bone mineral density may vary, with certain groups being more susceptible than others. However, due to the limited number of studies and lack of population-specific data, these findings cannot be generalized to the broader population. Genetic ancestry groups are rarely taken into account in studies investigating gene-phenotype associations, making it a common issue in such research studies.

Moreover, many failed to adequately control for confounding factors such as vitamin K intake, lifestyle factors, or concurrent medication use. This, together with the fact that none of the included studies performed multivariate analyses to adjust for multiple genetic variants or gene-environment interactions, significantly limits the understanding of the degree to which these SNPs actually impact BMD. This is an important issue, as BMD is a multifactorial phenotype influenced by both genetic and environmental factors.

Future research on larger and more diverse cohorts is needed to clarify the clinical value of these associations. Studies such as expression quantitative trait loci (eQTL) analyses could identify genetic variants that contribute to BMD. Furthermore, no data are currently available on whether vitamin K supplementation or higher doses can mitigate the negative effects associated with *VKORC1* risk alleles. Well-designed randomized controlled trials are required to address this knowledge gap.

## 5. Conclusions

A modest association was observed for the *VKORC1* rs9923231 variant among women. When considering all studies with rs9923231 and rs9934438 (as an indirect indicator of rs9923231 presence), a modest and significant effect of the risk alleles on osteopenia/osteoporosis was seen. However, these findings should be interpreted with caution given the small number of studies and limited sample sizes. Further studies are required to evaluate whether this impact is clinically meaningful in bone health.

## Data Availability

All data generated in this work is reported in the paper and Supplementary Materials. The corresponding author can be contacted for further information.

## Supplementary Materials

The following supporting information can be downloaded at https://www.mdpi.com/article/10.3390/medicina62010180/s1: Figure S1: Labeled Funnel plot (A) showing the publication bias analysis and Sunset enhanced Funnel plot (B) showing the individual power of the studies; Table S1: BMD measurement and osteoporosis diagnosis in each study; Table S2: Quality assessment results using the NOS scale.
