# Supplementary material for: Association Between VKORC1 Gene Polymorphisms and Osteopenia and Osteoporosis: A Systematic Review and Meta-Analysis
Source: Medicina (Kaunas). 2026 Jan 15;62(1):180. doi: 10.3390/medicina62010180 (PMC12843655; doi:10.3390/medicina62010180)
Supplement: Supplementary file 1 [file medicina-62-00180-s001.zip › medicina-3993598-supplementary.pdf]

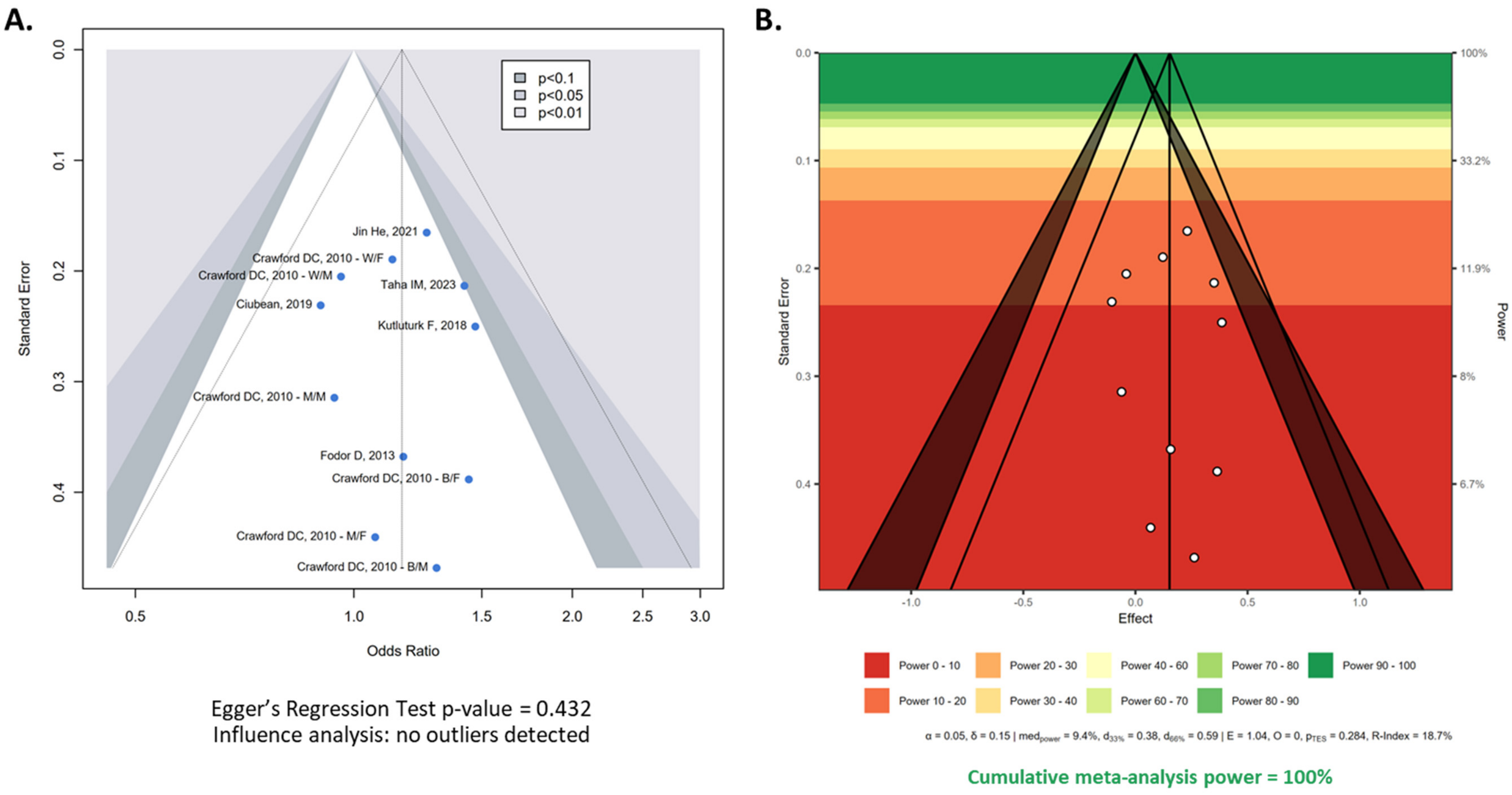

Supplementary Figure S1. Labeled Funnel plot (A) showing the publication bias analysis and Sunset enhanced Funnel plot (B) showing the individual power of the studies.

Supplementary Table S1. BMD measurement and osteoporosis diagnosis in each study.

**Author, year**	**anatomical measurement of BMD**	**criteria for osteoporosis/osteopenia**
Ciubean et al., 2019	Lumbar spine, Hip	not specified
Crawford et al., 2010	Proximal femur at the trochanter, intertrochanter, Ward's triangle and total region	-2.5 standard deviations from the mean BMD total region
Fodor et al., 2013	Lumbar spine, Femoral neck, Hip	T score below -2.5
Jin He, 2021	Lumbar spine	T score below -2.5
Kutluturk et al., 2018	Lumbar spine, Femoral neck	T score below -2.5
Taha et al., 2023	Femoral neck	T score below -2.5, negative Z score of 2

Supplementary Table S2. Quality assessment results using the NOS scale.

	**Selection**				**Comparability**	**Exposure**				
**Study**	**Q1.1**	**Q1.2**	**Q1.3**	**Q1.4**	**Q2**	**Q3.1**	**Q3.2**	**Q3.3**	**NOS total score**	**Quality**
Ciubean et al., 2019	1	1	1	1	2	1	1	1	**9**	High
Crawford et al., 2010	1	1	1	1	2	1	1	1	**9**	High
Fodor et al., 2013	1	1	1	1	1	1	1	1	**8**	High
Jin He, 2021	1	1	1	1	1	1	1	1	**8**	High
Kutluturk et al., 2018	1	1	1	1	1	1	1	1	**8**	High
Taha et al., 2023	1	0	1	1	1	1	1	1	**7**	Moderate–High
